# Association of Use of an Integrated Specialty Pharmacy With Total Medical Expenditures Among Members of an Accountable Care Organization

**DOI:** 10.1001/jamanetworkopen.2020.18772

**Published:** 2020-10-06

**Authors:** Apurv Soni, Brian S. Smith, Thomas Scornavacca, Bill McElnea, Alice Shakman, Eric Dickson, David D. McManus

**Affiliations:** 1 Department of Population and Quantitative Health Sciences, University of Massachusetts Medical School, Worcester; 2 Shields Health Solutions, Stoughton, Massachusetts; 3 Office of Clinical Integration, UMass Memorial Health Care, Worcester, Massachusetts; 4 Clinical Services, UMass Memorial Medical Center, Worcester, Massachusetts; 5 Department of Emergency Medicine, University of Massachusetts Medical School, Worcester; 6 Department of Cardiovascular Medicine, University of Massachusetts Medical School, Worcester

## Abstract

This cohort study examines the association of integrated specialty pharmacy use among members of a university hospital accountable care organization (ACO) with total medical expenditure.

## Introduction

Use of integrated specialty pharmacies within Accountable Care Organizations (ACOs) optimizes medication adherence, increases care coordination with physicians, and reduces medication-related adverse events.^1,2,3^ They may also decrease health care costs for patients because medication coordination and fulfillment could reduce adverse events and improve underlying conditions, which in turn decreases health care visits. We examined the association between the use of integrated specialty pharmacies and total medical expenditure (TME) among the members of the largest ACO in central Massachusetts.

## Methods

Data for this retrospective matched cohort study were extracted from the UMass Memorial Medicare ACO (UMMACO) from January 2016 through December 2018. Patients of all ages receiving care from a specialty department were assigned to the intervention group if they were enrolled in the UMMACO integrated specialty pharmacy at the start of the study period and the control group if they were not. Their status did not change throughout the study period. To account for baseline differences between the groups, patients were matched on age, sex, and level of care based on the UMMACO risk stratification model for care management. Stratification was determined by a committee within UMMACO that accounts for complexity of patient care, including readmission, emergency department utilization, postacute care, and chronic disease management. Patients were matched without replacement. The outcome was the per-member per-month costs (PMPM) of TME, which were calculated for each month during the study period. We used multilevel generalized linear models to estimate the association of integrated specialty pharmacy use and PMPM, allowing us to account for repeated measurements among patients. Postestimation calculations were made for difference-in-difference analysis and statistical significance was assessed at 95% confidence levels (eAppendix in the Supplement). Results were considered significant at *P* < .05 in 2-tailed tests.

Analysis for this study was done in December 2019 and was performed using Stata software, version 15 (StataCorp). This study followed the Strengthening the Reporting of Observational Studies in Epidemiology (STROBE) reporting guideline. The study was reviewed by the University of Massachusetts Medical School institutional review board, and was exempted from informed consent because it used secondary data analyses of deidentified data.

## Results

Patients enrolled in UMMACO who used the organization’s integrated specialty pharmacy were younger compare with those who did not (median [SD] age, 63 [12.8] years vs 70.6 [12.8] years; *P* = .01) (Table 1). Matching increased comparability between the 2 groups. After adjusting for comorbidities, PMPM were similar in 2016 but increased for patients who did not use the integrated specialty pharmacy while decreasing for those who did (Table 2). Costs decreased by $267 (95% CI, −$1586 to $1052) for those who did use integrated specialty pharmacy while increasing by $1007 (95% CI, $270 to $1743) for patients who did not. The difference of difference for average net savings of integrated specialty pharmacy users vs nonintegrated specialty pharmacy users was $1274; however, this difference was not statistically significant (95% CI, −$215 to $2764) for the sample in this study.

**Table 1.** Characteristics of Patients Enrolled in the UMass Memorial Medicare Accountable Care Organization Included in This Study, Based on Their Integrated Specialty Pharmacy Use

Characteristic	Total patients, No. (%) (N = 9302)	All members with identified specialty care					
		Unmatched			Matched		
		No. (%)		*P* value	No. (%)		*P* value
		Integrated (n = 120)	Nonintegrated (n = 2875)		Integrated (n = 120)	Nonintegrated (n = 182)	
Age, mean (SD), y	71.2 (12.4)	63.6 (12.8)	70.6 (12.1)	<.01	63.6 (12.8)	65.3 (14.0)	.29
Women	4911 (52.8)	72 (59.8)	1636 (56.9)	.50	72 (59.8)	112 (61.3)	.79
Level of care^a^							
1, least complex	1767 (19.0)	30 (25.0)	578 (20.1)	.45	30 (25.0)	42 (23.3)	.99
2	2707 (29.1)	27 (22.8)	633 (22.0)		27 (22.8)	40 (21.8)	
3	1991 (21.4)	18 (15.2)	552 (19.2)		18 (15.2)	29 (16.0)	
4	1488 (16.0)	18 (15.2)	546 (19.0)		18 (15.2)	31 (17.2)	
5, most complex	1349 (14.5)	26 (21.7)	566 (19.7)		26 (21.7)	39 (21.7)	
Target conditions, mean (SD), No.	1.9 (1.0)	2.0 (1.0)	2.0 (1.1)	.98	2.0 (1.0)	2.1 (1.1)	.42
Mental comorbidities, mean (SD), No.	2.0 (0.5)	2.0 (0.3)	1.9 (0.5)	.43	2.0 (0.3)	2.0 (0.4)	.99
PMPM, mean (SD), $^b^							
2016	1073 (1561)	1538 (1972)	1206 (1620)	.03	1538 (1972)	1466 (1974)	.76
2017	1716 (3133)	2017 (3492)	1935 (3143)	.78	2017 (3492)	2171 (3736)	.72
2018	1534 (3192)	1978 (2754)	2109 (3862)	.71	1978 (2754)	2665 (3404)	.07

Abbreviation: PMPM, per-member per-month costs.

^a^ Level of care was defined by the care management model of the accountable care organization and included patients’ demographic information and comorbidities.

^b^ PMPM estimates are rounded to the nearest US dollar.

**Table 2.** Mean PMPM for Users of Integrated vs Nonintegrated Specialty Pharmacy Among Patients Enrolled in the UMass Memorial Medicare Accountable Care Organization^a^

Year(s)	Integrated specialty pharmacy use, PMPM point estimate (95% CI), $		Cost difference point estimate (95% CI), $	Difference of cost estimate difference (95% CI), $
	No	Yes		
**Annual**				
2016	2052 (1113 to 2991)	2121 (304 to 3938)	−69 (−1563 to 1425)	NA
2017	2673 (1966 to 3379)	2132 (369 to 3895)	541 (−854 to 1936)	NA
2018	3059 (2347 to 3771)	1854 (137 to 3571)	1205 (−156 to 2567)	NA
**Year-over-year**				
2017 vs 2016	621 (−88 to 1329)	11 (−1316 to 1338)	NA	610 (−883 to 2103)
2018 vs 2017	386 (−181 to 953)	−278 (−1550 to 995)	NA	664 (−727 to 2056)
2018 vs 2016	1007 (270 to 1743)	−267 (−1586 to 1052)	NA	1274 (−215 to 2764)

Abbreviations: NA, not applicable; PMPM, per-member per-month costs.

^a^ Results based on multivariable multilevel generalized models after users and nonusers of integrated specialty pharmacy were adjusted for age, sex, and level of care, specialty of department providing care, and comorbidities.

## Discussion

Our findings suggest that integrated specialty pharmacy use by patients enrolled in UMMACO is associated with net savings of more than $1000 per month from 2016 to 2018 compared with matched counterparts within UMMACO who did not use an integrated specialty pharmacy. Although not statistically significant, the magnitude of health care savings is notable in the context of previous findings of savings of as little as $34 (95% CI, $15-$52) for Medicare ACO patients (in a 2016 study of 15 592 600 participants)^4^ and savings of $114 (95% CI, $50-$178) among clinically vulnerable populations participating in a Medicare ACO (in a 2015 study of 8 673 823 participants).^5^

The results from our study should be interpreted in the context of the limited number of patients who used the integrated specialty pharmacy in our sample and the focus on patients receiving specialty care. While patients’ ability to opt into the integrated specialty pharmacy was not conditioned on a health insurance plan, unobserved patient characteristics and preferences may inform their choice. We made attempts to reduce bias from confounding by matching on key variables and adjusting for comorbidities and type of specialty department providing care. However, additional analyses and future studies (ie, a randomized cluster trial) are needed to identify the savings attributable directly to integrated specialty pharmacy use. Matching on key covariates improved comparability but limited our sample size to patients who could be matched on those variables. Nevertheless, our findings underscore the potential of specialty pharmacies to reduce TME.^6^ In the current value-based care landscape, the ability to use data to guide strategic interventions and provide analysis is essential for any ACO or value-based program. Finding scalable interventions that provide the full constellation of success for patients, health care professionals, and ACOs is exceptionally difficult. The integration of specialty pharmacies into care management models of care delivery has the promise to fulfill this goal.

## Notes

Supplement. **eAppendix.** Detailed Description of Sample and Variable Definition
