# Supplementary material for: Association of Use of an Integrated Specialty Pharmacy With Total Medical Expenditures Among Members of an Accountable Care Organization
Source: JAMA Netw Open. 2020 Oct 6;3(10):e2018772. doi: 10.1001/jamanetworkopen.2020.18772 (PMC7539116; doi:10.1001/jamanetworkopen.2020.18772)
Supplement: Supplement. — eAppendix. Detailed Description of Sample and Variable Definition [file jamanetwopen-e2018772-s001.pdf]

Supplementary Online Content

Soni A, Smith BS, Scornavacca T, et al. Association of use of an integrated specialty pharmacy with total medical expenditures among members of an accountable care organization. *JAMA Netw Open*. 2020;3(10):e2018772. doi:10.1001/jamanetworkopen.2020.18772

eAppendix. Detailed Description of Sample and Variable Definition

This supplementary material has been provided by the authors to give readers additional information about their work.

© 2020 Soni A et al. *JAMA Network Open.*

Appendix I: Detailed description of sample and variable definition

This study included all members of UMass Memorial Medicare Accountable Care Organization (UMMACO) who were receiving care from a specialty department, regardless of their age. The list of clinics includes Cardiology, Cystic Fibrosis, Dermatology, Endocrinology, Fertility, Gastroenterology, Hepatitis C, Infectious Disease, Multiple Sclerosis, Neurology, Oncology, Pediatric Endocrinology, Pediatric Gastrointestinal, Pediatric Pulmonary, Pulmonary, Rheumatology, and Transplant medicine. Specialty-pharmacy services at UMass Memorial are available to patients that receive their care at specialty clinics prescribing outpatient self-administered specialty medications. Patients are offered the choice at UMass Memorial Medical center specialty clinics to receive their medication from an integrated specialty-pharmacy. Patients using the integrated specialty-pharmacy receive clinical pharmacist support that includes patient education around proper use, storage, administration, and disposal of medication. Additionally, the service provides evaluation of drug-drug and drug-disease interactions, monitoring for side effects and medication adherence support. Patients were excluded if they died or left specialty care during the study period.

Data was derived from the UMMACO and specialty-pharmacy database, which uses data from patient's electronic health records and intake forms. Total medical expenditure in the form of per-member-per-month costs were calculated for each month during the study period and average annual per-member-per-month cost was included

© 2020 Soni A et al. *JAMA Network Open.*

in the dataset for each patient. Patients who used integrated pharmacy were matched to those who do not use integrated pharmacy based on age (current age in years +/- 2 years), sex, and level of care. Matching was performed without replacement and ties were included. We considered type of specialty, number of target conditions, and mental comorbidities as potential confounders. Target conditions were problems that were identified by a specialty clinic for which the patient was receiving medical care. We did not include number of visits as a confounder because it could be in the pathway of how integrated pharmacy use is associated with total medical care expenditure i.e. integrated specialty-pharmacy use could reduce number of visits by improving medication adherence and preventing adverse effects of medications leading to lesser number of visits.

Association between integrated specialty-pharmacy use and per-member-per-month trends from 2016-18 were assessed using multilevel generalized linear models that accounted for repeated measurement among patients across years. Taylor linearization methods were used to calculate differences and difference of differences between the integrated and non-integrated users and standard error was estimated using the delta method in Stata 15. In addition to adjusting for the matching factors, estimates were adjusted for target conditions, mental comorbidities, and type of specialty.

© 2020 Soni A et al. *JAMA Network Open.*
